# Acknowledgement to Reviewers of *Viruses* in 2014

**DOI:** 10.3390/v7010110

**Published:** 2015-01-12

**Authors:** Viruses Editorial Office

**Affiliations:** MDPI AG, Klybeckstrasse 64, CH-4057 Basel, Switzerland

The editors of *Viruses* would like to express their sincere gratitude to the following reviewers for assessing manuscripts in 2014:

Abram, Michael
Accotto, Gian Paolo
Ackermann, Hans-Wolfgang
Aertsen, Abram
Afonso, Claudio L.
Agranovsky, Alexey
Ahlm, Clas
Akula, Shaw M.
Alemany, Ramon
Allain, Jean-Pierre
Allison, W. Ted
Almazan, Fernando
Alonso, Marta M.
Amari, Khalid
An, Dong Jun
An, Zhiqiang
Anderson, Jeffrey P.
Anderson, Paul
Anfoka, Ghandi
Aquaro, Stefano
Arai, Satoru
Arbiza, Juan
Arhel, Nathalie J.
Arikawa, Jiro
Arnaud, Frederick
Awasthi, Sita
Babiuk, Shawn
Bachmann, Martin
Badley, Andrew
Bagamian, Karoun
Bajorek, Monika
Bálint, Ádám
Banfield, Bruce
Bannert, Norbert
Bányai, Krisztián
Banyard, Ashley
Bárcena, Montserrat
Barlow, John
Barry, Michael
Barry, Peter
Bartenschlager, Ralf
Bartha, István
Bartlett, Paul C.
Bartosch, Birke
Benfield, David A.
Benhenda, Shirine
Beniac, Daniel R.
Benko, Mária
Bergthaler, Andreas
Bernard, Kristen
Berniak, Hanna
Bernstein, David I.
Bershteyn, Anna
Bessong, Pascal
Bhardwaj, Neeru
Biegalke, Bonita
Biegalke, Bonita J
Black, William
Blair, Carol
Blanco, José Luis
Blasdel, Robert
Blass, Dieter
Blitvich, Bradley
Blomström, Anne-Lie
Bochkov, Yury A.
Bodewes, Rogier
Boudinot, Pierre
Bourhy, Hervé
Bowen, Richard
Bradfute, Steven
Branton, Philip E.
Branza-Nichita, Norica
Bresnahan, Wade
Briddon, Rob
Brinton, Margo
Broccolo, Francesco
Brockman, Mark A.
Broder, Christopher C.
Brody, Thomas
Bruggeman, Leslie
Brune, Wolfram
Bugert, Joachim
Buller, Robert Mark Laidlaw
Burand, John
Calisher, Charles
Cameron, Craig
Card, J. Patrick
Cardin, Rhonda
Cardoso, Tereza C.
Carvalho, R.F.
Casasnovas, José M.
Caughey, Byron
Cen, Osman
Chae, Chanhee
Chan, Gary
Chandran, Kartik
Chang, Ruey-Yi
Charpentier, Charlotte
Chejanovsky, Nor
Chen, Benjamin
Chen, Jason J.
Chen, Ji-Ming
Chen, Xulin
Chen, Ying-hua
Chernomordik, Leonid
Chiu, Wah
Choi, Seung-Kook
Choi, Young-Ki
Chomont, N
Chow, Louise T.
Christophides, George
Cingolani, Gino
Ciota, Alex
Clark, Jason
Claverie, Jean-Michel
Clouser, Christine
Cobbs, Charles
Cocquerel, Laurence
Coffin, John M.
Cohrs, Randall
Colebunders, Robert
Connolly-Andersen, Anne-Marie
Conrad, Nicholas
Cooke, Brian
Cosby, Louise
Costa, Luciana
Coulibaly, Fasseli
Csorba, Tibor
Cui, Jie
Curiel, David
Das, Kalyan
Dash, Srikanta
de Geus, Eveline D.
de La Torre, Juan Carlos
de Los Santos, Teresa
de Marco, Federico
de Noronha, Carlos M.C.
de Vries, Rory
Debyser, Zeger
Dee, Scott
DeFilippis, Victor R.
Denner, Joachim
Desbiez, Cécile
DeStefano, Jeffery
Diamond, Michael
Díaz Pendón, Juan Antonio
Diehl, William
Dietzgen, Ralf G
Dimitrov, Dimiter
Dittmer, Dirk
Dobner, Thomas
Drebot, Mike
Drulis-Kawa, Zuzanna
Dubuisson, Jean
Duerksen-Hughes, Penelope J.
Dutch, Rebecca
Dutilh, Bas
Ebel, Greg
Echevvaria, Juan
Eckerle, Isabella
Eisenstark, Abraham
El Zowalaty, Mohamed
Elder, John H.
Elliott, Richard M.
Emerson, Ginny
Enric Mateu, Enric
Erlandson, Martin
Erskine, Ronald J.
Esclatine, Audrey
Essbauer, Sandra
Esteban, M.
Evans, Matthew
Everett, Gabby
Fair, Jeanne
Fang, Ying
Feldmann, Heinz
Feng, Pinghui
Fernandes, Patricia
Fichet-Calvet, E.
Figueiredo, Luiz Tadeu
Finley, Russell L.
Firth, Cadhla
Fish, Eleanor
Fletcher, Simon
Foley, Brian T.
Fooks, Anthony R.
Fortunato, Elizabeth
Fox, Manon MJ
Fredericksen, Brenda L.
Freed, Eric
Freiberg, Alexander N.
Frenkel, Lisa
Freuling, Conrad
Friesen, Paul D.
Fuchs, Marc
Fuentes, Alejandro
Fujihashi, Kohtaro
Fukushi, Hideto
Gabuzda, Dana
Gallay, Phillip
Garcia, Pilar
Garten, Wolfgang
Gayral, Philippe
Geballe, Adam P.
Geisbert, Thomas
Georgel, Philippe
Ghanim, Murad
Giam, Chou-Zen
Gifford, Robert
Gilch, Sabine
Goepfert, Paul
Goff, Stephen P.
Goldfarb, Samuel
Goldmana, Ellen R.
Goldsmith, Cynthia
Golovanov, Alexander P.
Gompels, Ursula
Goodrum, Felicia
Grandvaux, Nathalie
Greber, Urs F.
Greenlee, Justin J.
Gregory, James
Groseth, Allison
Gubler, Duane
Gupta, Rashmi
Hadidi, Ahmed
Haegeman, Andy
 
Haisma, Hidde
Halford, William
Hall, Alex R.
Hall, Roy A.
Hampson, Ian N.
Han, Guan-zhu
Han, Yeon Soo
Hannah, Matthew J.
Harrach, Balazs
Harris, David A.
Harrison, Robert
Hartshorn, Kevan
Harty, Ronald N.
Hauber, Joachim
Hauton, Chris
Hayasaka, Daisuke
Hayman, David
Hefferon, Kathleen Laura
Hegyi, Hedi
Heldwein, Ekaterina
Henderson, Andrew
Henzy, Jamie
Hepojoki, Jussi
Herath, Tharangani
Hernalsteens, Jean-Pierre
Hill, Andrew F.
Hinkula, Jorma
Hirsch, Alec J.
Hirsch, Vanessa
Hjelle, Brian
Hoeben, Rob C.
Hoenen, Thomas
Holbrook, Michael R.
Holmes, Kay
Honko, Anna
Horiuchi, Motohiro
Houpt, Eric R.
Hruby, Dennis
Huang, Jinghe
Huang, Ning
Huckriede, Anke
Hudson, Amy
Hughes, Grant
Hughes, Joseph
Hur, Mina
Iordanskiy, Sergey
Iorio, Ronald M.
Izumiya, Yoshi
J. Kuhn, Richard
Jacobs, Bertram
Jacobson, Ken
Jakobs, Stefan
Jan, Fuh-Jyh
Jehle, Johannes
Jenkins, Frank
Jenson, Vicki
Jeske, Holger
Jones, Ian
Jonsson, Colleen
Jørgensen, Jorunn B.
Kalejta, Robert
Kamen, Amine
Kamil, Prof., Jeremy P.
Kamp, Christel
Kariwa, Hiroaki
Karlsson, Erik
Kashanchi, Fatah
Kassiotis, George
Kaufer, Benedikt
Kennedy, Richard G.
Kerry, Julie
Kesmir, Can
Khatri, Mahesh
Khromykh, Alexander
Kikuchi, Yutaka
Kim, Chul-Joong
Kindrachuk, Jason
Kitchen, Andrew
Klein, Christian
Klempa, Boris
Klingström, Jonas
Knobler, Charles
Knox, Caroline
Kobinger, Gary
Kohl, Alain
Kohl, Claudia
Koma, Takaaki
Komar, Nicholas
Kondo, Hidehiro
König, Matthias
Korch, George
Köster, Mario
Krammer, Florian
Kranzusch, Philip
Krogstad, Paul
Kropinski, Andrew
Ku, Hsin-Mei
Kuenzi, Amy
Kuhn, Jens
Kuhn, Richard
Kumar, Mukesh
Kumar, Sachin
Kumar-Singh, Rajendra
Kuritzkes, Daniel R.
Labo, Nazzarena
Lager, Kelly M.
Laimins, Laimonis A.
Lamien, Charles Euloge
Lan, Ke
Landini, Maria Paola
Landry, Nathalie
Lasmézas, Corinne Ida
Lau, Susanna
Lawson, Victoria A.
Le Grice, Stuart Jf
Lecollinet, Sylvie
Lee, Benhur
Leibowitz, Julian
Lemmerman, Niels
Leroux, Caroline
Leventhal, Jeremy
Li, Feng
Li, Mei-Ling
Li, Zheng
Liang, Jake
Lichty, Brian
Linial, Maxine L.
Lippé, Roger
Liu, Shan-Lu
Liu, Wen
Liu, Zhijun
Livieratos, Ioannis C.
Lloyd, Richard E.
Lowenstein, Pedro
Lu, Wuxun
Ludlow, Martin
Lukac, David
Lukashevich, Igor
Lundström, Jan
Lundstrom, Kenneth
MacFarlane, Stuart A.
Mackay, Ian
Mackow, Erich
MacNeill, Amy
Mager, Dixie
Main, Roger
Majerciak, Vladimir
Mak, Johnson
Marche, Patrice N.
Margolis, David
Markovitz, David
Marques, Joao
Marsh, Glenn
Martín-Acebes, Miguel A.
Martinez-Sobrido, Luis
Massimelli, Julia
Maury, Wendy
McCormick, Craig
McElhinney, Lorraine
McIlroy, Dorian
McVoy, Michael
Medina, Rafael A.
Mehle, Andrew
Melero, José A.
Menendez-Arias, Luis
Mercer, Jason
Meredith, Anna
Mertens, Thomas
Meyerett-Reid, Crystal
Michiels, Thomas
Miller, Christopher J.
Miller, Patti J.
Miller, William
Millhauser, Glenn
Milligan, Gregg
Miyazawa, Takayuli
Mlotshwa, Sizolwenkosi
Mocarski, Edward
Mochizuki, Tomofumi
Mohr, Ian
Monjane, Adérito L
Monk, Peter
Montgomery, JoLynn P.
Montgomery, Ruth R
Moog, Christiane
Morales, R.
Morello, Chris
Morgan, Robin
Morikawa, Yuko
Morrison, Richard
Morrison, Trudy G
Moseley, Gregory
Moss, Bernard
Moss, Paul
Mühlebach, Michael
Muljono, DH
Munk, Carsten
Munro, Sandra B.
Murk, Jean-Luc
Muyldermans, Serge
Naffakh, Nadia
Nagata, Noriyo
Nair, Venugopal
Nefedova, L.N.
Nemerow, Glen R.
Neumann, Peter
Neven, Lisa G.
Nichol, Stuart
Nie, Xianzhou
Niedrig, Matthias
Niedźwiedzka-Rystwej, Paulina
Nikolaidis, Nikolas
Nikolopoulos, Georgios
Nishizono, Akira
Noris, Emanuela
Nowotny, Norbert
Nowrouzi, Ali
Oglesbee, Michael
Ohishi, Kazue
Ohki, Takehiro
Ohlmann, Théophile
Olson, Kenneth
Ono, Akira
Oomens, Antonius
Oren, Kobiler
Overbaugh, J.
Padmanabhan, R.
Paeshuyse, Jan
Paintsil, Elijah
Palese, Peter
Pallas, Vicente
Palmarini, Massimo
Paprotka, Tobias
Pari, Gregory
Parish, Jo
Park, Bong-Kyun
Parks, Robin
Parrish, Colin
Pata, Janice
Patel, Arvind
Pathak, Vinay
patil, basavaprabhu
Peeters, Ben P. H.
Peng, Zong Gen
Perreault, Jean-Pierre
Pessler, Frank
Petrini, Stefano
Pfeffer, Sébastien
Pfeifer, Blaine
Pfeiffer, Julie K.
Piganeau, Gwenaël
Pijlman, Groben
Pintó, Rosa M.
Pleschka, Stephan
Plyusnin, Alexander
Poehlmann, Stefan
Pöhlmann, Stefan
Poli, Guido
Polonsky, Jonathan A.
Polyak, Stephen J.
Possee, Robert
Powers, Ann M.
Prado, Julia
Prescott, Joseph
Puchhammer-Stöckl, Elisabeth
Qiu, Hua-Ji
Quax, Tessa
Rabinowitz, Peter M.
Ramasamy, Selvarajan
Rappocciolo, Giovanna
Ray, Stuart
Reddy, Vijay
Reeves, Matthew
Reeves, Roger Keith
Regeard, Christophe
Reid, Steven
Rein, Alan
Reina, Ramses
Renault, Tristan
Renne, Rolf
Rethwilm, Axel
Rice, Andrew
Rima, Bert
Risco, Cristina
Rohr, Olivier
Roizman, Bernard
Romalde, Jesús López
Romero, Carlos
Rong, Lijun
Roques, Pierre
Rose, Nicola
Rose, Timothy M.
Rosenthal, Ken
Rowland, Raymond
Roye, Marcia
Rubenstein, Richard
Ruiz-Fons, José Francisco
Ryu, Wang-Shick
Sagan, Selena
Saleh, Maria-Carla
Sambrie, Vittorio
Sánchez-Madrid, Francisco
Sang, Yongming
Santangelo, Philip J.
Saphire, Erica
Sather, Noah
Sauer, Markus
Scagnolari, Carolina
Schildgen, Oliver
Schirrmacher, Volker
Schmaljohn, Connie
Schmidt, Manfred
Schountz, Tony
Schubert, Jörg
Schug, Alexander
Schwegmann, Christel
Segundo, Fayna Diaz-San
Semmes, Oliver John
Séron, Karin
Serracca, Laura
Serwer, Philip
Seto, Donald
Setoh, Yin
Shai, Yechiel
Shannon-Lowe, Claire
Shoukry, Naglaa
Siddell, Stuart
Siewe, Basile
Silva, Jerson L.
Silvie, Olivier
Simmen, Thomas
Simón, Oihane
Sinclair, John
Singh, Sudhir
Sinkins, Steven
Sironen, Tarja
Sluis-Cremer, Nicolas
Smartt, Chelsea T.
Smith, Darci
Smith, David W.
Smith, Ina
Smith, Kellie N
Söderhäll, Kenneth
Söderlund Strand, Anna
Somia, Nik
Song, Jin-Won
Soothill, James
Sorokulova, Iryna
Spatz, Steve J.
Spearman, Paul
Spencer, Juliet
Spilki, Fernando
Spindler, Katherine R.
Spiropoulou, Christina
Stamminger, Thomas
Stedman, Kenneth
Steinmann, Joerg
Stephens, Edward
Steven, Alasdair C.
Stockley, Peter
Stone, Daniel
Stoye, Jonathan
Strick, Reiner
Strive, Tanja
Strong, James
Summerfield, Artur
Supattapone, Surachai
Sutter, Gerd
Suzuki, Ryosuke
Suzuki, Tohru
Svoboda, Jan
Switzer, William
Takeda, Makoto
Takeuchi, Yasuhiro
Tamerius, James D.
Tang, Hengli
Tang, Liang
Tang, Shuang
Tang, Yiwei
Tarakanova, Vera L.
Taylor, Geraldine
Taylor, Matthew
Taylor, Shannon
Terajima, Masanori
Terrier, Olivier
Terzieva, Vesilava
Teschke, Carolyn
Thiel, Volker
Thomas, Christopher
Tischler, Nicole
Tomaras, Georgia D.
Tordo, Noel
Torres-Perez, Fernando
Toth, Karoly
Trobridge, Grant D.
Tsukiyama-Kohara, Kyoko
Turner, Stephen
Uldrick, Thomas
Ulrich, Rainer
Utaisincharoen, P
Valles, Steven
Valverde, Rodrigo A.
Van der Kuyl, Antoinette
Van Duyne, Rachel
Van Engelenburg, Schuyler
Van Lint, Carine
Vander Hoek, Lia
Vapalahti, Olli
Verjans, Georges M.G.M.
Verma, Subhash C
Vieillard, Vincent
Viejo-Borbolla, Abel
Visner, Gary
von Hahn, Thomas
von Messling, Veronika
Walker, Peter J
Wallis, Carole
Wanasen, Nanchaya
Wang, Linding
Wang, Xiaowei
Wang, Xiuqing
Ward, Vernon K.
Warrilow, David
Watashi, Koichi
Watts, Douglas
Webby, Richard
Weingartl, Hana M.
Weitzman, Matthew
Whitby, Denise
White, Laura
White, Stephen
Whittaker, Gary R.
Wiethoff, Christopher M.
Wills, Mark
Wolff, Dana
Woo, Soo Dong
Wood, Charles
Wu, Hung-Yi
Wu, Min
Wu, Ting-Ting
Xi, Zhiyong
Xu, Wenxing
Yanez-Mo, Maria
Yang, De
Yang, Zengqi
Yoder, Kristine
Yoon, Kyoung-Jin
Yoshiyama, Hironori
You, Jianxin
Young, Joanne
Yu, Qingzhong
Yu, Xuejie
Zakhartchouk, Alexander
Zhang, Tiejun
Zheng, Zhi-Ming
Zhirnov, Oleg
Zhu, Hua
Zhu, Huanzhang
Ziegelbauer, Joseph

